# Oncologic immunomodulatory agents in patients with cancer and COVID-19

**DOI:** 10.1038/s41598-021-84137-5

**Published:** 2021-03-01

**Authors:** Justin Jee, Aaron J. Stonestrom, Sean Devlin, Teresa Nguyentran, Beatriz Wills, Varun Narendra, Michael B. Foote, Melissa Lumish, Santosha A. Vardhana, Stephen M. Pastores, Neha Korde, Dhwani Patel, Steven Horwitz, Michael Scordo, Anthony F. Daniyan

**Affiliations:** 1 Department of Medicine, Memorial Sloan Kettering Cancer Center, 1275 York Avenue, New York, NY 10065 USA; 2 Department of Epidemiology and Biostatistics, Memorial Sloan Kettering Cancer Center, New York, NY USA; 3 Cancer Biology and Genetics Program, Memorial Sloan Kettering Cancer Center, New York, NY USA; 4 Department of Medicine, Weill Cornell Medical College, New York, NY USA; 5 Department of Anesthesiology and Critical Care Medicine, Memorial Sloan Kettering Cancer Center, New York, NY USA

**Keywords:** Medical research, Cancer

## Abstract

Corticosteroids, anti-CD20 agents, immunotherapies, and cytotoxic chemotherapy are commonly used in the treatment of patients with cancer. It is unclear how these agents affect patients with cancer who are infected with SARS-CoV-2. We retrospectively investigated associations between SARS-CoV-2-associated respiratory failure or death with receipt of the aforementioned medications and with pre-COVID-19 neutropenia. The study included all cancer patients diagnosed with SARS-CoV-2 at Memorial Sloan Kettering Cancer Center until June 2, 2020 (N = 820). We controlled for cancer-related characteristics known to predispose to worse COVID-19 as well as level of respiratory support during corticosteroid administration. Corticosteroid administration was associated with worse outcomes prior to use of supplemental oxygen; no statistically significant difference was observed in sicker cohorts. In patients with metastatic thoracic cancer, 9 of 25 (36%) and 10 of 31 (32%) had respiratory failure or death among those who did and did not receive immunotherapy, respectively. Seven of 23 (30%) and 52 of 187 (28%) patients with hematologic cancer had respiratory failure or death among those who did and did not receive anti-CD20 therapy, respectively. Chemotherapy itself was not associated with worse outcomes, but pre-COVID-19 neutropenia was associated with worse COVID-19 course. Relative prevalence of chemotherapy-associated neutropenia in previous studies may account for different conclusions regarding the risks of chemotherapy in patients with COVID-19. In the absence of prospective studies and evidence-based guidelines, our data may aid providers looking to assess the risks and benefits of these agents in caring for cancer patients in the COVID-19 era.

## Introduction

The practical management of Coronavirus Disease 2019 (COVID-19), caused by severe acute respiratory syndrome coronavirus 2 (SARS-CoV-2), and management of cancer in SARS-CoV-2 endemic regions remains a major challenge for oncologists. Severe COVID-19 is characterized by a profound and uncontrolled pro-inflammatory cytokine response that can lead to lung injury and death^1^. However, the risk profile of immunomodulatory agents commonly used in standard cancer management is not established in patients with COVID-19. Recent studies have identified cancer patient characteristics, such as thoracic and hematologic malignancy, associated with worse outcomes, but there is mixed data regarding the outcomes of cancer patients with COVID-19 treated with immunomodulatory agents^2–8^.

For instance, the World Health Organization (WHO) and Infectious Disease Society of America (IDSA) initially recommended against the routine use of corticosteroids in patients with COVID-19, in part due to concerns that they may decrease viral clearance, and only recently did the IDSA suggest corticosteroid use in hospitalized patients with severe COVID-19 based on new evidence of potential benefit in noncancer patients^9–12^. Corticosteroids are common components of cancer treatment and supportive care. However, their impact on cancer patients with COVID-19 is not well studied. A recent retrospective study found a possible trend toward worse outcomes associated with corticosteroid use in cancer patients, although no analysis was performed to correct for possible selection bias in which sicker patients received those medications^11^.

Recent evaluations of the impact of cytotoxic chemotherapy on COVID-19 outcomes have found differing results^4,5,7,8^. Although in a 309-patient study, we found no evidence of worse outcomes in patients who had received recent cytotoxic chemotherapy in general, we did find that pre-COVID-19 neutropenia was associated with worse COVID-19 outcomes^7^. Further analysis regarding the interactions between neutropenia and chemotherapy at the time were precluded by sample size. Observational studies have also reported variable results regarding the impact of immunotherapy (i.e. PD-1 and CTLA-4 blockade) on COVID-19 outcomes^5,6,13–15^. Anti-CD20 monoclonal antibodies are commonly used in treatment of hematologic malignancy and autoimmune diseases, but there is little data regarding the impact of these agents on COVID-19 course.

To address this dearth of data, we performed a retrospective analysis of the potential effects of commonly used immunomodulatory agents on cancer patients with COVID-19.

## Methods

### Patients

The study included all adult cancer patients followed at Memorial Sloan Kettering Cancer Center (MSKCC) who developed COVID-19 in the inpatient or outpatient setting between March 8, 2020 and June 2, 2020. SARS-CoV-2 status was determined using a nasopharyngeal swab to determine the presence of virus specific RNA (MSKCC FDA EUA-approved assay and Cephied®). Clinical outcomes were monitored until June 3, 2020. All patient data was obtained from the MSKCC electronic medical record. The MSKCC institutional review board approved the study and waived the requirement for informed consent. All research was performed in accordance with relevant guidelines and regulations.

### Data sources

Patient data was extracted from the MSKCC electronic medical record (EMR). Patient medications, demographics, laboratory measurements, and outcomes (i.e. use of supplemental oxygen, noninvasive or invasive mechanical ventilation, and death), were extracted from a standardized-input institutional database. Relevant comorbidities as well as thoracic and hematologic cancer status were extracted from International Classification of Disease (ICD)-9 and ICD-10 codes using a process cross-validated with manual curation^7^.

### Statistical analysis

For all analyses we considered the number of patients who developed a primary composite endpoint of respiratory failure (use of nonrebreather, high-flow nasal oxygen, or mechanical ventilation) or death within 28 days of SARS-CoV-2 diagnosis. Differences in rates of event between groups in the anti-CD20, immunotherapy, and neutropenia cohorts were assessed by Fisher’s exact test.

### High-dose corticosteroids

We evaluated the impact of corticosteroid administration (defined as the equivalent of at least 60 mg cumulative of prednisone over COVID-19 course) on the composite outcome. In an attempt to partially control for selection bias in which more acutely ill patients received corticosteroids with therapeutic intent, we performed a series of multivariate Cox proportional hazards analyses with hematologic malignancy and corticosteroid use as variables. Corticosteroid administration was introduced as a time-dependent variable. We performed a “pre-supplemental oxygen” analysis from time of SARS-CoV-2 diagnosis to an endpoint of first use of > 2 L/min supplemental oxygen or death. We also performed a “post-supplemental oxygen” analysis from a start time of first use of > 2 L/min supplemental oxygen to an endpoint of respiratory failure or death. This model included only patients who had received > 2 L/min supplemental oxygen. The 2L/min cutoff was chosen to cohort patients with similar levels of COVID-19 severity at time of corticosteroid administration. We also performed a “post-critical” analysis from a start time of first use of nonrebreather, high-flow nasal oxygen, or mechanical ventilation to an endpoint of death. All patient events for these analyses were right-censored at 28 days following SARS-CoV-2 diagnosis or June 3, 2020, whichever came first. Hazard ratios (HR) and 95% confidence intervals (CI) were reported for these analyses.

### Anti-CD20 therapy

We estimated the incidence in composite outcome in patients receiving anti-CD-20 therapy (rituximab, ofatumumab, or obinutuzumab within 90 days prior to SARS-CoV-2 diagnosis). Because patients with hematologic malignancy are more likely to receive these agents, we also estimated the rate of respiratory failure or death just among patients with hematologic malignancy.

### Immunotherapy

We estimated the incidence of the composite outcome in patients receiving immunotherapy (PD-1, PDL-1, or CTLA-4 blockade within 90 days prior to SARS-CoV-2 diagnosis). This was additionally estimated separately among patients with a history of thoracic malignancy and among patients with metastatic disease and who had types of cancer that might make them eligible for immunotherapy, as performed by previous studies^6^.

### Neutropenia

We evaluated the association of “pre-COVID-19 neutropenia” with the composite outcome. Pre-COVID-19 neutropenia was defined as any absolute neutrophil count, ANC < 1 K/µL between 7 and 60 days prior to SARS-CoV-2 diagnosis. We also performed analyses stratified based on whether or not a given patient received cytotoxic chemotherapy in the same time frame and whether or not the patient had a neutropenic measurement within 30 days of chemotherapy administration. We also considered whether or not a given patient had a diagnosis of hematologic malignancy. We separately examined incidences of respiratory failure or death in patients with “mild neutropenia,” i.e. at least one recorded ANC < 2 K/µL between 7 and 60 days prior to SARS-CoV-2 diagnosis but no recorded ANC < 1 K/µL in that timeframe. We also examined incidences of respiratory failure or death in patients with “recovered neutropenia,” i.e. at least one ANC < 1 K/µL in a given time window prior to SARS-CoV-2 diagnosis but not subsequently. For the “recovered neutropenia” analysis, two time windows were considered: 7 to 60 days and, separately, 60 to 180 days prior to SARS-CoV-2 diagnosis.

We compared interleukin-6 (IL-6), C-reactive protein (CRP), ferritin, and white blood cell (WBC) levels prior to and after high-dose corticosteroid administration using Wilcoxon signed-rank tests. Only values seven days prior to or after medication administration were considered. In the case of repeat laboratory measurements within the seven-day window the value closest to medication administration was used. We also compared those laboratory measurements within three days of SARS-CoV-2 diagnosis between patients who did and did not receive prior immunotherapy using Mann–Whitney U tests.

All analyses were performed using Python 3.7.4 and the “lifelines” package version 0.22.8.

## Results

A total of 820 cancer patients were diagnosed with SARS-CoV-2 during the study period. Patient characteristics are reported in Table 1. Collinearity between these characteristics is explored in Figure S1. A total of 761 patients (92.8%) were followed for the maximum possible time of 28 days or died. A total of 382 patients (46.5%) were admitted, 154 (18.8%) reached the primary composite endpoint, and 97 (11.8%) died.

**Table 1:** Patient characteristics (not mutually exclusive).

	Number	% total
Total	820	100.0
Age > 60	451	55.0
Male	396	48.3
White	506	61.7
Black or African American	143	17.4
Asian	70	8.5
Other	64	7.8
Unknown	37	4.5
Smoking history	313	38.2
≥ 3 comorbidities	332	40.5
BMI > 30	230	28.0
Neutropenia	61	7.4
Thoracic cancer	74	9.0
Hematologic cancer	210	25.6
Solid metastatic cancer	332	40.5
First corticosteroid prior to critical illness^a^	30	3.7
First corticosteroid during critical illness^b^	54	6.6
Prior immunotherapy	51	6.2
Prior anti-CD20	26	3.2
Received > 2 L/min supplemental oxygen	162	19.8
Reached composite endpoint	154	18.8
Died	97	11.8

^a^High-flow oxygen, ventilation, or death.

^b^During high-flow oxygen or ventilation and prior to death.

Patients with a history hematologic malignancy were more likely to receive corticosteroids (Figure S1). Among patients with hematologic malignancy, 15 patients received corticosteroids, seven (48%) of whom developed respiratory failure or died. By contrast 52 of 195 (27%) patients with hematologic malignancy not receiving steroids reached that endpoint. These medications were also administered with higher levels of supplemental oxygen relative to patients who did not develop respiratory failure or died (Figure S2). When patients were stratified by level of respiratory support, corticosteroid use was associated with worse outcomes in the pre-2 L oxygen cohort (HR 2.3, 95% CI 1.1–4.9), a trend not observed in the post-2 L oxygen (HR 0.9, 95% CI 0.4–1.9) and post-critical cohorts (HR 0.8, 95% CI 0.5–1.4), though these additional analyses were limited by the sample size (Table S1). Indications for corticosteroid administration in the pre-hypoxic setting are reported in Table S2; there was no clear link between steroid indication and outcomes. Five of 17 (29%) patients who received high-dose corticosteroids in the pre-hypoxic setting had a prior standing prescription for corticosteroids at the time of COVID-19 diagnosis; of those five patients, two ultimately utilized > 2 L/min oxygen. IL-6 and CRP levels were lower, while ferritin levels were higher, after corticosteroid administration in both the pre and post-critical illness settings (Figure S3).

Nine of 26 patients (35%) treated with prior anti-CD20 agents had respiratory failure or died; although this was higher than the rate of respiratory failure or death in the cohort as a whole, the rates based on prior anti-CD20 agents were similar in the subset of patients with hematologic cancer (Table 2). The rates of respiratory failure or death based on prior immunotherapy were estimated in both the subset of patients with metastatic thoracic cancer and the subset of other metastatic solid cancers that were eligible for immunotherapy (Table 2). Nine of 25 patients (35%) with thoracic metastatic cancer who received immunotherapy developed respiratory failure or died, compared with 10 of 31 (32%) patients with thoracic metastatic cancer who did not receive immunotherapy. Immunotherapy administration was not associated with elevated IL-6 and CRP at time of SARS-CoV-2 diagnosis (Figure S3).

**Table 2:** Rate respiratory failure or death among patients treated and not treated with anti-CD20 agents and immunotherapy (IO) and with or without neutropenia pre-COVID-19.

	# resp. failure or death/total in category		p-value
	Received anti-CD20	No anti-CD20	
Total	9/26 (35%)	145/794 (18%)	0.03
Hematologic	7/23 (30%)	52/187 (28%)	> 0.1
Solid	2/3 (66%)	93/607 (15%)	> 0.1

	Received IO	No IO	
Total	14/51 (27%)	140/769 (18%)	> 0.1
Thoracic (all)	9/26 (35%)	12/48 (25%)	> 0.1
Other solid (all)	4/23 (17%)	79/511 (15%)	> 0.1
Thoracic (metastatic)	9/25 (36%)	10/31 (32%)	> 0.1
Selected solid (metastatic)^a^	3/18 (17%)	18/101 (18%)	> 0.1

	Neutropenia	No neutropenia	
Total	24/61 (39%)	130/759 (17%)	0.0001
On cytotoxic chemotherapy	14/38 (37%)	33/180 (18%)	0.02
No chemotherapy	10/23 (43%)	97/579 (17%)	0.003
Hematologic	15/37 (41%)	44/173 (25%)	0.07
Solid	9/24 (38%)	86/586 (15%)	0.006
Mild neutropenia^b^	18/107 (17%)	112/652 (17%)	> 0.1
7–60 days pre-COVID, recovered^c^	15/35 (43%)	139/785 (18%)	0.0007
60–180 days pre-COVID, recovered	4/24 (17%)	150/796 (19%)	> 0.1

	Received corticosteroid	No corticosteroid	
Total	10/30 (33%)	144/790 (18%)	0.05
Hematologic	7/15 (47%)	52/195 (27%)	> 0.1
Solid	3/15 (20%)	92/595 (15%)	> 0.1

^a^Limited to patients with solid metastatic cancer deemed eligible for immunotherapy by cancer type (N = 179), specifically triple negative breast cancer, colorectal cancer, renal cancer, bladder cancer, melanoma, cervical or uterine cancer, esophageal or stomach cancer, or hepatocellular carcinoma.

^b^Mild neutropenia: at least one measured ANC < 2 K/µL and all measured ANC > 1 K/µL within 7 to 60 days prior to SARS-CoV-2 diagnosis.

^c^Recovered neutropenia: at least one measured ANC < 1 K/µL during the stated time window and subsequently at least one ANC ≥ 1 K/µL and no measurements of ANC < 1 K/µL after SARS-CoV-2 diagnosis.

Cytotoxic chemotherapy administration seven to 60 days prior to SARS-CoV-2 diagnosis was not associated with worse outcomes; however, patients who developed neutropenia following cytotoxic chemotherapy had worse outcomes. Of 38 patients with chemotherapy-associated pre-COVID-19 neutropenia, 14 (37%) developed respiratory failure or died. Patients with neutropenia unrelated to chemotherapy also had worse outcomes, regardless of solid or hematologic cancer type (Table 2). Of note, patients with neutropenia seven to 60 days prior to SARS-CoV-2 diagnosis but had “recovered” from neutropenia also had worse outcomes. Patients who had recovered from neutropenia 60 to 180 days prior to SARS-CoV-2 diagnosis did not have worse outcomes. Patients with mild neutropenia only (ANC < 2 K/µL) did not have worse outcomes (Table 2).

Of note, four patients had pre-COVID-19 neutropenia and were treated with immunotherapy; of these four patients, three developed respiratory failure or died.

## Discussion

We present a single-center, retrospective analysis of cancer patients with COVID-19 treated with various immunomodulatory agents. Our study demonstrates that in assessing whether certain oncologic medications are associated with worse COVID-19 outcomes, considering cancer type, degree of effect (i.e. neutropenia or other bone marrow suppression) and other patient-specific factors is crucial.

Studies in non-cancer patients have suggested that corticosteroids may have a role in early COVID-19 illness^16^. In the cohort of patients treated with corticosteroids prior to use of > 2 L/min supplemental oxygen, we found a trend toward worse outcomes, although this may reflect selection bias for patients suspected at risk for decompensation receiving these medications. There is also promising evidence that corticosteroids may reduce mortality in critically ill patients with COVID-19-related respiratory failure^12,17^. We did not observe a decrease in adverse events in critically ill patients^12^, although our cohort sizes for these subsets were limited. Further study on the impact of corticosteroids on critically ill cancer patients is needed. The clinical significance of lower IL-6 and CRP levels and higher ferritin levels following corticosteroid use in cancer patients also requires further inquiry.

We did not observe worse outcomes in patients receiving anti-CD20 therapy when cancer type was considered. The rate of respiratory failure or death was somewhat lower than in a smaller cohort of multiple sclerosis patients treated with rituximab, although that study also did not find worse COVID-19 outcomes with disease modifying agents as a whole^18^.

We found that although cytotoxic chemotherapy itself was not a risk for worse outcomes, pre-COVID-19 neutropenia was associated with worse COVID-19. A minority of patients in our cohort receiving cytotoxic chemotherapy developed neutropenia; differences in the proportion of patients developing neutropenia while receiving chemotherapy may account for why previous studies have found both absence^4,5^ and presence^8^ of harm with these agents. Previous trends toward worse COVID-19 outcomes with thrombocytopenia at baseline and during infection have also been observed^7^. At the same time, abnormally high ANC has also been observed during in certain patients with worse COVID-19^7^. It is plausible that bone marrow suppression or other immunosuppressive features, rather than neutropenia per se, are responsible for the worse outcomes seen in the neutropenic cohort of our study. It is noteworthy that even patients who may be recovering from relatively recent neutropenic events were found to have worse COVID-19 outcomes. Although it is reassuring that more distant neutropenia and milder neutropenia were not associated with adverse outcomes, the relationship between recovery from immunosuppression and COVID-19 warrants further exploration.

The observed rates of respiratory failure or death were 36% and 32% for metastatic lung cancer patients who did and did not receive immunotherapy, respectively. We utilized a definition of metastatic disease that includes only solid tumors with distal or widespread metastases which may in part account for the variable findings compared to previous report in a similar population^6^. Immunotherapy use, thoracic cancer, smoking history, and metastatic solid cancer are highly associated (Figure S1), making it difficult to truly understand which are truly responsible for worse COVID-19 outcomes. Evaluation of the specific risks of immunotherapy use in patients with COVID likely requires larger analyses in cancer-specific cohorts.

It is also notable that of patients with pre-COVID-19 neutropenia treated with immunotherapy, three of four developed respiratory failure or died. All of these patients had solid metastatic disease and were treated with either prior or concomitant cytotoxic chemotherapy. Although recent chemotherapy is currently not believed to be a risk factor for worse COVID-19 outcomes, it has been observed that patients receiving combination chemotherapy and immunotherapy may have higher rates of severe respiratory COVID-19^7^. The potential interaction between antineoplastic agents as well as cancer severity and COVID-19 warrants further study.

Despite efforts to control for potential confounders, this study has several limitations inherent to retrospective research. Because of the modest number of patients treated with most of the medications in question, more complex multivariate models were not performed. Although we controlled for lung and hematologic cancers, studies including patients overlapping with those in this cohort suggest that certain hematologic cancer subtypes may predispose to better or worse outcomes^7,19–21^. Certain immunomodulatory agents could not be included due to sparse representation. For example, 12 patients in our cohort received intravenous immunoglobulin; five of these patients developed respiratory failure or died. Inferring the harm or benefit of medications such as corticosteroids in a retrospective study is inherently biased. It is plausible that sicker patients were more likely to receive corticosteroids, and that this was not captured by our study variables. Many risk factors for COVID-19 are unknown, and COVID-19 treatment patterns are complex. It is certain that both patient baseline and COVID-19 related confounders exist in our dataset. Randomized, prospective cancer-specific clinical trials are needed to evaluate the safety and effectiveness of these agents in treating cancer patients with COVID-19.

## Supplementary Information

Supplementary Information.

## Data Availability

The datasets generated during and/or analysed during the current study are available from the corresponding author on reasonable request.
