# Supplementary material for: Oncologic immunomodulatory agents in patients with cancer and COVID-19
Source: Sci Rep. 2021 Mar 1;11:4814. doi: 10.1038/s41598-021-84137-5 (PMC7921444; doi:10.1038/s41598-021-84137-5)
Supplement: Supplementary file 1 — Supplementary Information. [file 41598_2021_84137_MOESM1_ESM.docx]

Supplemental Information for “Oncologic Immunomodulatory Agents in Patients with Cancer and COVID-19”

Justin Jee, Aaron J. Stonestrom, Sean Devlin, Teresa Nguyentran, Beatriz Wills, Varun Narendra, Michael B. Foote, Melissa Lumish, Santosha Vardhana, Stephen M. Pastores, Neha Korde, Dhwani Patel, Steven Horwitz, Michael Scordo, Anthony Daniyan

|  | 1. Pre-supplemental oxygen | | | 2. Post-supplemental oxygen | | | 3. Post-critical | | |
| --- | --- | --- | --- | --- | --- | --- | --- | --- | --- |
|  | HR | - 95%CI | + 95%CI | HR | - 95%CI | + 95%CI | HR | - 95%CI | + 95%CI |
| Hematologic cancer | 1.6 | 1.2 | 2.0 | 1.4 | 0.9 | 2.0 | 0.9 | 0.5 | 1.4 |
| Corticosteroid event | 2.3 | 1.1 | 4.9 | 0.9 | 0.4 | 1.9 | 0.8 | 0.5 | 1.4 |

Table S1. HR and 95% CI in multivariate analysis for 1. Analysis of time from SARS-CoV-2 diagnosis to use of >2L/min supplemental oxygen or death and 2. Analysis from time of >2L/min supplemental oxygen use to need for high-flow oxygen, intubation, or death. 3. Analysis from time of high-flow oxygen or intubation to death.

| Steroid Indication | No event (N=10) | Event (N=7) | Total (N = 17) |
| --- | --- | --- | --- |
| COVID-19 treatment | 2 | 3 | 5 |
| Suspected adrenal insufficiency | 3 | 2 | 5 |
| Cancer treatment | 1 | 2 | 3 |
| Immunotherapy toxicity | 1 | 0 | 1 |
| Other | 3 | 0 | 3 |

Table S2. Indications for high-dose corticosteroid use in the pre-supplemental oxygen setting. “Event” signifies progression to utilize >2L/min supplemental oxygen.


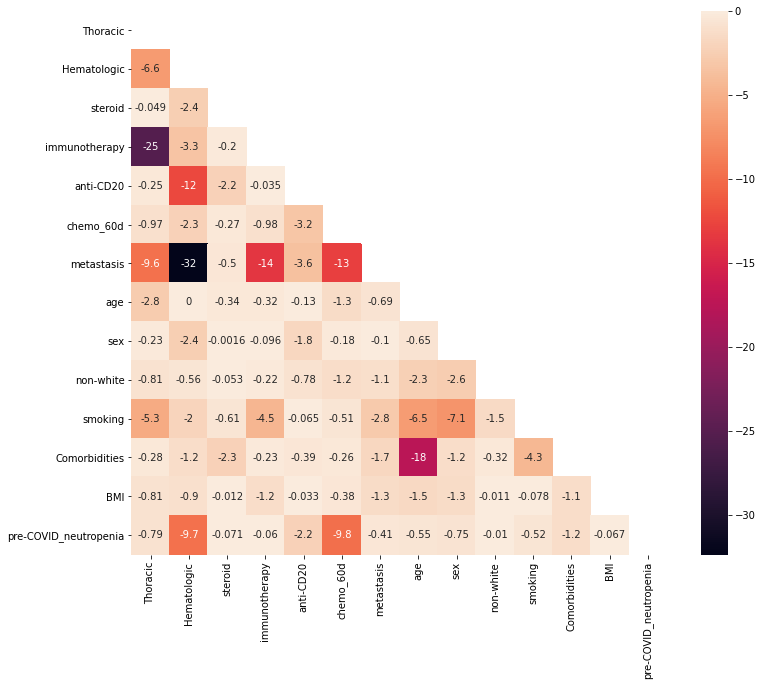


Figure S1. Pairwise Chi-square testing among variables. Values shown are log_10_(p-value). chemo_60d = cytotoxic chemotherapy within 60 days of SARS-CoV-2 diagnosis.


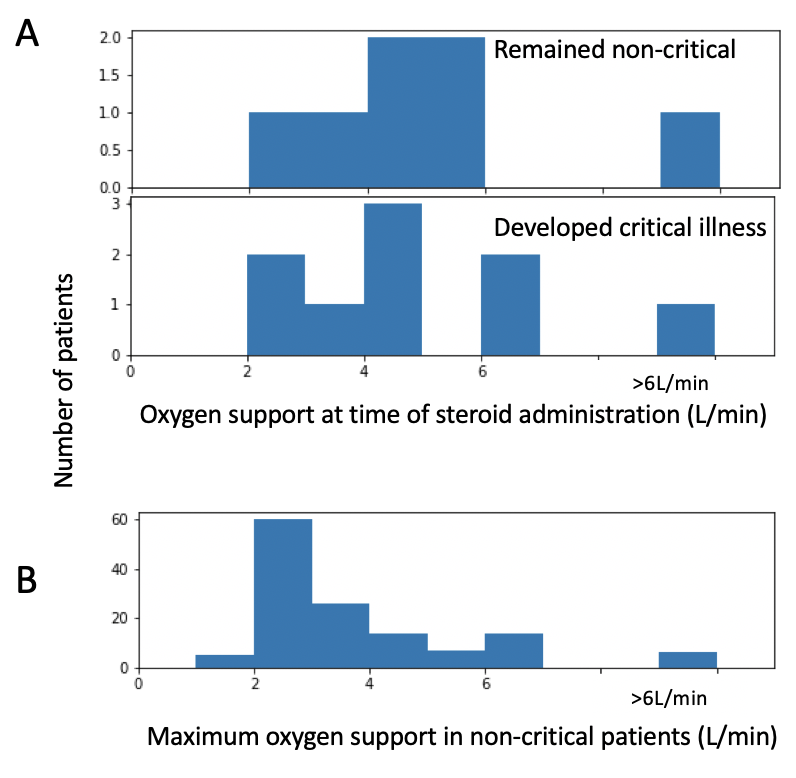


Figure S2. A. Histogram of amount of oxygen (L/min) a given patient was on when receiving high-dose corticosteroids, separated into patients who developed a requirement for high-flow oxygen or mechanical ventilation or died (“Developed critical illness”) or did not (“Remained non-critical”). B. Histogram of maximum oxygen support in all patients who utilized supplemental oxygen but did not develop critical illness. Patients who did not require oxygen or who ultimately received high-flow oxygen or mechanical ventilation are excluded.


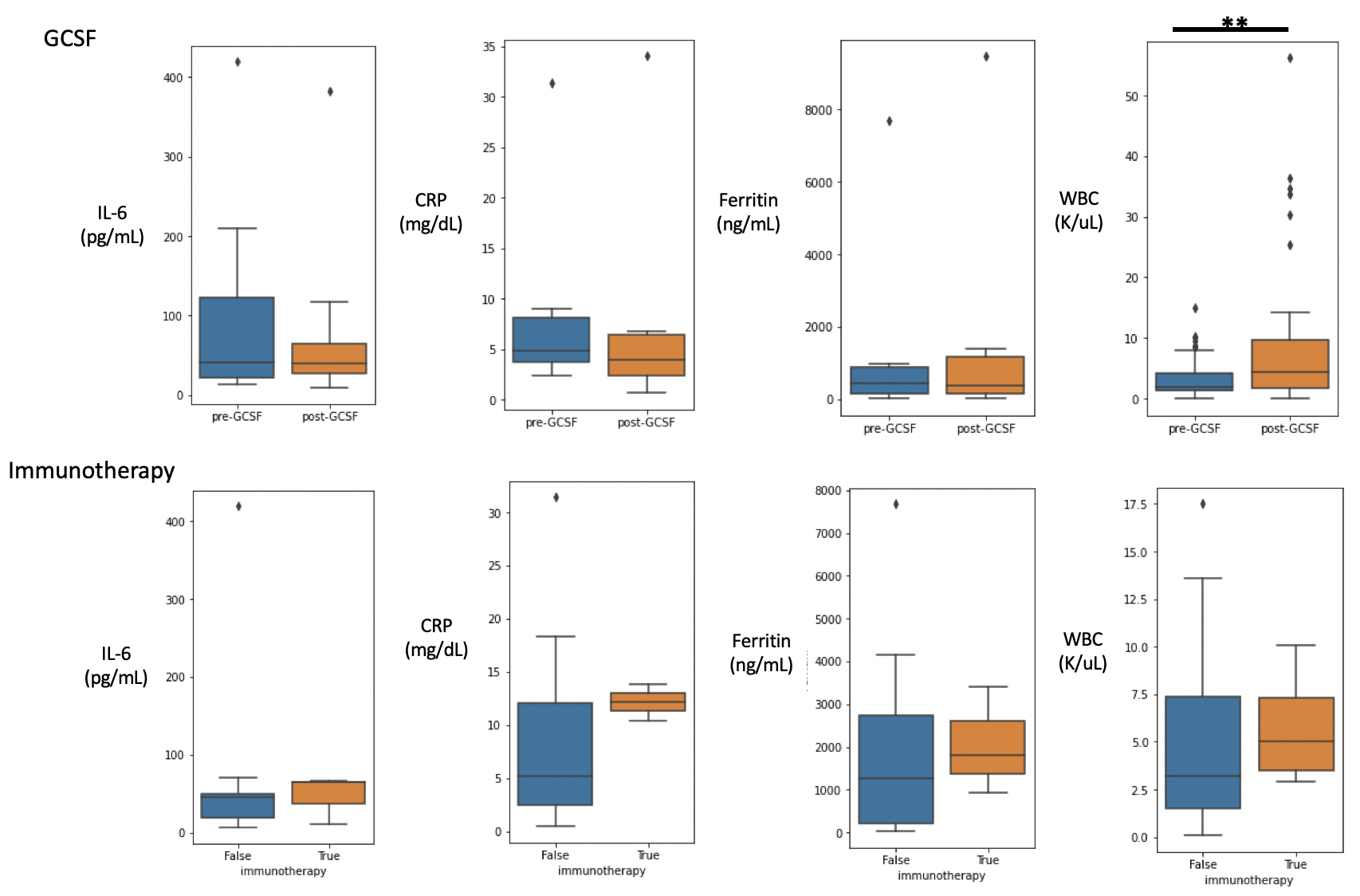


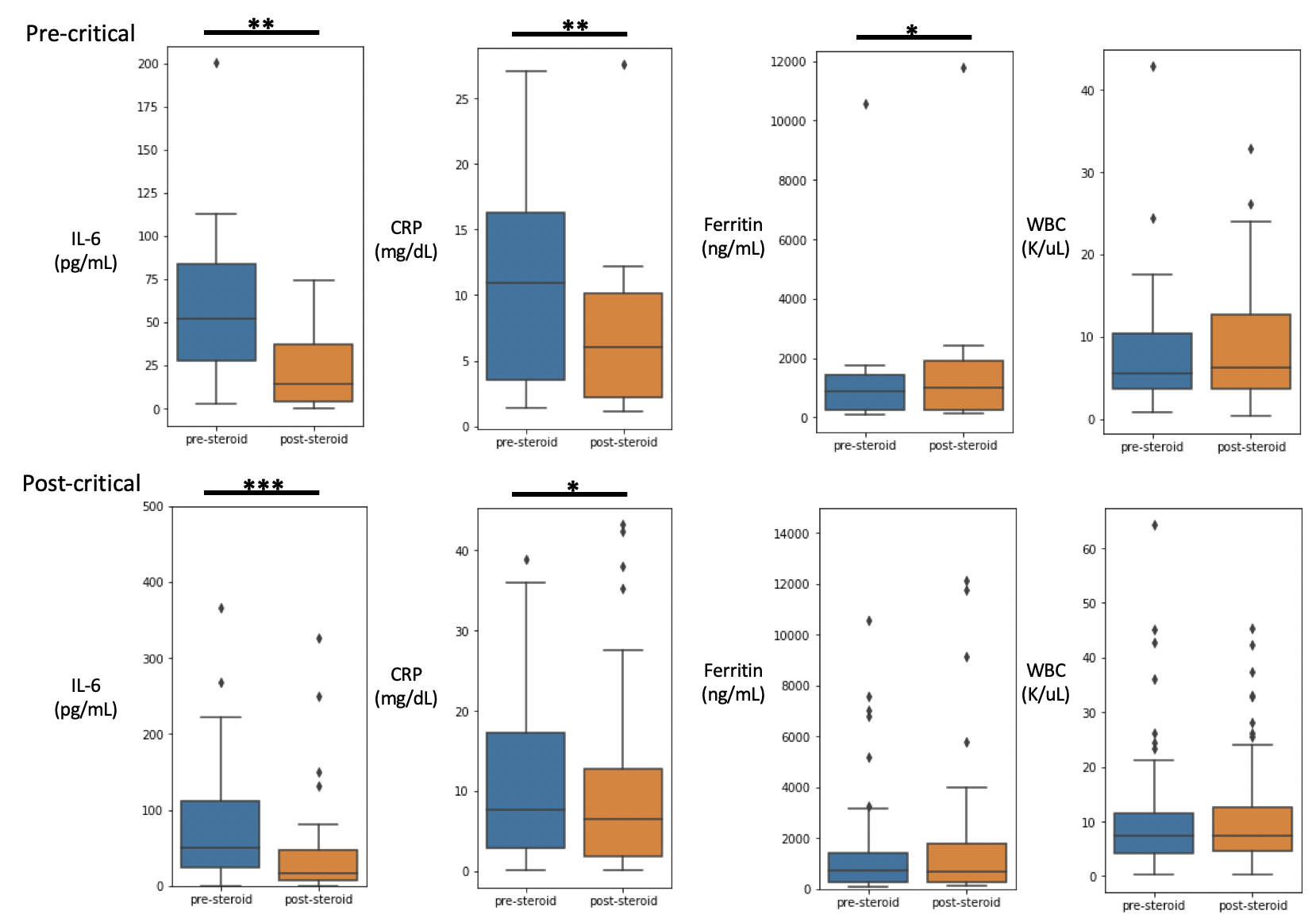


Figure S3. (Top) Boxplot showing quartiles and outliers of earliest laboratory values within 3 days of SARS-CoV-2 diagnosis for patients treated or not treated with immunotherapy 90 days prior. No comparisons were significant by Mann-Whitney U test with p-value cutoff of 0.05. Below, boxplots showing quartiles with outliers of paired laboratory values from prior to and after administration of high-dose corticosteroids (middle) prior to respiratory failure (nonrebreather, high-flow nasal oxygen or mechanical ventilation) and (bottom) after respiratory failure. In the post-critical IL-6 plot, outliers >500pg/mL are excluded. In the post-critical ferritin plot, outliers > 15,000ng/mL are excluded. *** p< 0.0001, ** p<0.001, * p<0.01 by Wilcoxon signed-rank test.
